# Deep learning for chest radiograph diagnosis: A retrospective comparison of the CheXNeXt algorithm to practicing radiologists

**DOI:** 10.1371/journal.pmed.1002686

**Published:** 2018-11-20

**Authors:** Pranav Rajpurkar, Jeremy Irvin, Robyn L. Ball, Kaylie Zhu, Brandon Yang, Hershel Mehta, Tony Duan, Daisy Ding, Aarti Bagul, Curtis P. Langlotz, Bhavik N. Patel, Kristen W. Yeom, Katie Shpanskaya, Francis G. Blankenberg, Jayne Seekins, Timothy J. Amrhein, David A. Mong, Safwan S. Halabi, Evan J. Zucker, Andrew Y. Ng, Matthew P. Lungren

**Affiliations:** 1 Department of Computer Science, Stanford University, Stanford, California, United States of America; 2 Department of Medicine, Quantitative Sciences Unit, Stanford University, Stanford, California, United States of America; 3 Department of Radiology, Stanford University, Stanford, California, United States of America; 4 Department of Radiology, Duke University, Durham, North Carolina, United States of America; 5 Department of Radiology, University of Colorado, Denver, Colorado, United States of America; Edinburgh University, UNITED KINGDOM

## Abstract

**Background:**

Chest radiograph interpretation is critical for the detection of thoracic diseases, including tuberculosis and lung cancer, which affect millions of people worldwide each year. This time-consuming task typically requires expert radiologists to read the images, leading to fatigue-based diagnostic error and lack of diagnostic expertise in areas of the world where radiologists are not available. Recently, deep learning approaches have been able to achieve expert-level performance in medical image interpretation tasks, powered by large network architectures and fueled by the emergence of large labeled datasets. The purpose of this study is to investigate the performance of a deep learning algorithm on the detection of pathologies in chest radiographs compared with practicing radiologists.

**Methods and findings:**

We developed CheXNeXt, a convolutional neural network to concurrently detect the presence of 14 different pathologies, including pneumonia, pleural effusion, pulmonary masses, and nodules in frontal-view chest radiographs. CheXNeXt was trained and internally validated on the ChestX-ray8 dataset, with a held-out validation set consisting of 420 images, sampled to contain at least 50 cases of each of the original pathology labels. On this validation set, the majority vote of a panel of 3 board-certified cardiothoracic specialist radiologists served as reference standard. We compared CheXNeXt’s discriminative performance on the validation set to the performance of 9 radiologists using the area under the receiver operating characteristic curve (AUC). The radiologists included 6 board-certified radiologists (average experience 12 years, range 4–28 years) and 3 senior radiology residents, from 3 academic institutions. We found that CheXNeXt achieved radiologist-level performance on 11 pathologies and did not achieve radiologist-level performance on 3 pathologies. The radiologists achieved statistically significantly higher AUC performance on cardiomegaly, emphysema, and hiatal hernia, with AUCs of 0.888 (95% confidence interval [CI] 0.863–0.910), 0.911 (95% CI 0.866–0.947), and 0.985 (95% CI 0.974–0.991), respectively, whereas CheXNeXt’s AUCs were 0.831 (95% CI 0.790–0.870), 0.704 (95% CI 0.567–0.833), and 0.851 (95% CI 0.785–0.909), respectively. CheXNeXt performed better than radiologists in detecting atelectasis, with an AUC of 0.862 (95% CI 0.825–0.895), statistically significantly higher than radiologists' AUC of 0.808 (95% CI 0.777–0.838); there were no statistically significant differences in AUCs for the other 10 pathologies. The average time to interpret the 420 images in the validation set was substantially longer for the radiologists (240 minutes) than for CheXNeXt (1.5 minutes). The main limitations of our study are that neither CheXNeXt nor the radiologists were permitted to use patient history or review prior examinations and that evaluation was limited to a dataset from a single institution.

**Conclusions:**

In this study, we developed and validated a deep learning algorithm that classified clinically important abnormalities in chest radiographs at a performance level comparable to practicing radiologists. Once tested prospectively in clinical settings, the algorithm could have the potential to expand patient access to chest radiograph diagnostics.

## Abstract 2

In their study, Pranav Rajpurkar and colleagues test a deep learning algorithm that classifies clinically important abnormalities in chest radiographs.

## Author summary

**Why was this study done?:**

- Chest radiographs are the most common medical imaging test in the world and critical for diagnosing common thoracic diseases.
- Radiograph interpretation is a time-consuming task, and there is shortage of qualified trained radiologists in many healthcare systems.
- Deep learning algorithms that have been developed to provide diagnostic chest radiograph interpretation have not been compared to expert human radiologist performance.

**What did the researchers do and find?:**

- We developed a deep learning algorithm to concurrently detect 14 clinically important pathologies in chest radiographs.
- The algorithm can also localize parts of the image most indicative of each pathology.
- We evaluated the algorithm against 9 practicing radiologists on a validation set of 420 images for which the majority vote of 3 cardiothoracic specialty radiologists served as ground truth.
- The algorithm achieved performance equivalent to the practicing radiologists on 10 pathologies, better on 1 pathology, and worse on 3 pathologies.
- Radiologists labeled the 420 images in 240 minutes on average, and the algorithm labeled them in 1.5 minutes.

**What do these findings mean?:**

- Deep learning algorithms can diagnose certain pathologies in chest radiographs at a level comparable to practicing radiologists on a single institution dataset.
- After clinical validation, algorithms such as the one presented in this work could be used to increase access to rapid, high-quality chest radiograph interpretation.

## Introduction

Chest radiography is the most common type of imaging examination in the world, with over 2 billion procedures performed each year [1]. This technique is critical for screening, diagnosis, and management of thoracic diseases, many of which are among the leading causes of mortality worldwide [2]. A computer system to interpret chest radiographs as effectively as practicing radiologists could thus provide substantial benefit in many clinical settings, from improved workflow prioritization and clinical decision support to large-scale screening and global population health initiatives.

Recent advancements in deep learning and large datasets have enabled algorithms to match the performance of medical professionals in a wide variety of other medical imaging tasks, including diabetic retinopathy detection [3], skin cancer classification [4], and lymph node metastases detection [5]. Automated diagnosis from chest imaging has received increasing attention [6,7], with specialized algorithms developed for pulmonary tuberculosis classification [8,9] and lung nodule detection [10], but the use of chest radiographs to discover other pathologies such as pneumonia and pneumothorax motivates an approach that can detect multiple pathologies simultaneously. Only recently have the computational power and availability of large datasets enabled the development of such an approach. The National Institutes of Health’s release of ChestX-ray14 led to many more studies that use deep learning for chest radiograph diagnosis [11–13]. However, the performance of these algorithms has not been compared to that of practicing radiologists.

In this work, we aimed to assess the performance of a deep learning algorithm to automatically interpret chest radiographs. We developed a deep learning algorithm to concurrently detect the presence of 14 different disease classes in chest radiographs and evaluated its performance against practicing radiologists.

## Methods

### Data

The ChestX-ray14 dataset [14] was used to develop the deep learning algorithm. The dataset is currently the largest public repository of radiographs, containing 112,120 frontal-view (both posteroanterior and anteroposterior) chest radiographs of 30,805 unique patients. Each image in ChestX-ray14 was annotated with up to 14 different thoracic pathology labels that were chosen based on frequency of observation and diagnosis in clinical practice. The labels for each image were obtained using automatic extraction methods on radiology reports, resulting in 14 binary values per image, where 0 indicates the absence of that pathology and 1 denotes the presence (multiple pathologies can be present in each image). We partitioned the dataset into training, tuning, and validation (see S1 Table for statistics of dataset splits used in this study).

The training set was used to optimize network parameters, the tuning set was used to compare and choose networks, and the validation set was used to evaluate CheXNeXt and radiologists. There is no patient overlap among the partitions.

### Radiologist annotations

A validation set of 420 frontal-view chest radiographs was selected from ChestX-ray14 for radiologist annotation. The set was curated to contain at least 50 cases of each pathology according to the original labels provided in the dataset by randomly sampling examples and iteratively updating the selected examples by sampling from the examples labeled with the underrepresented pathologies. The radiographs in the validation set were annotated by 3 independent board-certified cardiothoracic specialist radiologists (average experience 15 years, range 5–28 years) for the presence of each of the 14 pathologies. The majority vote of their annotations was taken as a consensus reference standard on each image. To compare to the algorithm, 6 board-certified radiologists from 3 academic institutions (average experience 12 years, range 4–28 years) and 3 senior radiology residents also annotated the validation set of 420 radiographs for all 14 labels. All radiologists individually reviewed and labeled each of the images using a freely available image viewer with capabilities for picture archiving and communication system features such as zoom, window leveling, and contrast adjustment. Radiologists did not have access to any patient information or knowledge of disease prevalence in the data. Labels were entered into a standardized data entry program, and the total time to complete the review was recorded. The Stanford International Review Board (IRB) approved this study, and all radiologists consented to participate in the labeling process.

### Algorithm development

The deep learning algorithm, called CheXNeXt, is a neural network trained to concurrently detect the 14 pathologies in frontal-view chest radiographs. Neural networks are functions with many parameters that are structured as a hierarchy of layers to model different levels of abstraction. In this study, the selected architecture was a convolutional neural network, a particular type of neural network that is specially designed to handle image data. By exploiting a parameter sharing receptive field, convolutional neural networks scan over an image to learn features from local structure and aggregate the local features to make a prediction on the full image. The neural network used in this study is a 121-layer DenseNet architecture [15] in which each layer is directly connected to every other layer within a block. For each layer, the feature maps of all preceding layers are used as inputs, and its own feature maps are passed on to all following layers as inputs.

Once specifying the neural network architecture, the parameters are automatically learned from a large amount of data labeled with the presence or absence of each pathology. The learning process consists of iteratively updating the parameters to decrease the prediction error, which is computed by comparing the network’s prediction to the known annotations on each image. By performing this procedure using a representative set of images, the resulting network can make predictions on previously unseen frontal-view chest radiographs.

### Training procedure

The training process consisted of 2 consecutive stages to account for the partially incorrect labels in the ChestX-ray14 dataset. First, multiple networks were trained on the training set to predict the probability that each of the 14 pathologies is present in the image. Then, a subset of those networks, each chosen based on the average error on the tuning set, constituted an ensemble that produced predictions by computing the mean over the predictions of each individual network. The ensemble was used to relabel the training and tuning sets as follows: first, the ensemble probabilities were converted to binary values by computing the threshold that led to the highest average F1 score on the tuning set across all pathologies. Then, the new label was taken to be positive if and only if either the original label was positive or the ensemble prediction was positive. Finally, new networks were trained on the relabeled training set, and a subset of the new networks was selected based on the average error on the relabeled tuning set. The final network was an ensemble of 10 networks trained on the relabeled data, where again the predictions of the ensemble were computed as the mean over the predictions of each individual network.

Before both stages of training, the parameters of each network were initialized with parameters from a network pretrained on ImageNet [16]. The final fully connected layer of the pretrained network was replaced with a new fully connected layer producing a 14-dimensional output, after which the sigmoid was applied to each of the outputs to obtain the predicted probabilities of the presence of each of the 14 pathology classes. Before inputting the images into the network, the images were resized to 512 pixels by 512 pixels and normalized based on the mean and standard deviation (SD) of images in the ImageNet training set. For each image in the training set, a random lateral inversion was applied with 50% probability before being fed into the network. The networks were updated to minimize the sum of per-class weighted binary cross entropy losses, where the per-class weights were computed based on the prevalence of that class in the training set. All parameters of the networks were trained jointly using Adam with standard parameters [17]. Adam is an effective variant of an optimization algorithm called stochastic gradient descent, which iteratively applies updates to parameters in order to minimize the loss during training. We trained the networks with minibatches of size 8 and used an initial learning rate of 0.0001 that was decayed by a factor of 10 each time the loss on the tuning set plateaued after an epoch (a full pass over the training set). In order to prevent the networks from overfitting, early stopping was performed by saving the network after every epoch and choosing the saved network with the lowest loss on the tuning set. No other forms of regularization, such as weight decay or dropout, were used. Each stage of training completed after around 20 hours on a single NVIDIA GeForce GTX TITAN Black. Each network had 6,968,206 learnable parameters, and the final ensemble had 69,682,060 parameters.

The open-source deep learning framework PyTorch (http://pytorch.org/) was used to train and evaluate the algorithms.

### Interpreting network predictions

In order to interpret predictions, CheXNeXt produced heat maps that identified locations in the chest radiograph that contributed most to the network’s classification through the use of class activation mappings (CAMs) [18]. To generate the CAMs, images were fed into the fully trained network, and the feature maps from the final convolutional layer were extracted. A map of the most salient features used in classifying the image as having a specified pathology was computed by taking the weighted sum of the feature maps using their associated weights in the fully connected layer. The most important features used by CheXNeXt in its prediction of the pathology were identified in the image by upscaling the map to the dimensions of the image and overlaying the image.

### Statistical analysis and evaluation on the validation set

We provide a comprehensive comparison of the CheXNeXt algorithm to practicing radiologists across 7 performance metrics, namely, area under the receiver operating characteristic curve (AUC), sensitivity, specificity, F1 metric, positive and negative predictive value (PPV and NPV), and Cohen’s kappa [19]. To convert the probabilities produced by CheXNeXt to binary predictions, we chose pathology-specific thresholds through maximization of the F1 score on the tuning set (more details presented in S1 Appendix).

To compare the CheXNeXt algorithm to radiologists using a single diagnostic performance measure, we used the AUC metric. Because the radiologists only provided yes/no responses for each image and not a continuous score, the receiver operating characteristic (ROC) was estimated for the radiologists as a group using partial least-squares regression with constrained splines to fit an increasing concave curve to the specificities and sensitivities of 9 radiologists. We specify knots at each 1/20th and assume symmetry. An example with R code is provided in S1 Appendix.

Because we estimate the ROCs for the radiologists, we cannot use standard confidence intervals (CIs) for the radiologists' AUCs, and so to ensure a fair comparison, we calculated and compared the respective AUCs in the same manner, as follows. We first estimate the ROC for the radiologists using constrained splines—as described above—and the ROC for the algorithm and then estimate the AUCs for both the algorithm and the radiologists using linear interpolation and the composite trapezoidal rule. Finally, we use the robust bootstrap method, described below, to construct CIs around the AUCs.

In addition to individual-level and pathology-specific performance measures, the CheXNeXt algorithm was evaluated over all pathologies and against radiologists as a group. To evaluate CheXNeXt against resident radiologists as a group and board-certified radiologists as a group, the micro-averages of the performance measures were computed across all resident radiologists as well as across all board-certified radiologists. Micro-averages for groups of radiologists were calculated by concatenating the predictions of group members and then calculating the performance measures. For example, to calculate the sensitivity for board-certified radiologists in predicting hernia (420 images), we concatenated each of 6 board-certified radiologists' predictions into a single array of length 420 × 6 = 2,520, repeated the reference standard for hernia 6 times to create an array of the same length, and then calculated sensitivity. To provide an overall estimate of accuracy, the proportion correct was calculated for each image across all 14 pathologies, and the mean and SD of these proportions are reported.

The nonparametric bootstrap was used to estimate the variability around each of the performance measures; 10,000 bootstrap replicates from the validation set were drawn, and each performance measure was calculated for CheXNeXt and the radiologists on these same 10,000 bootstrap replicates. This produced a distribution for each estimate, and the 95% bootstrap percentile intervals (2.5th and 97.5th percentiles) are reported [20].

Because AUC is a single measure on which to compare the CheXNeXt algorithm to the radiologists as a group, the difference between the AUCs on these same bootstrap replicates was also computed. To control the familywise error rate when testing for significant differences in AUCs, the stringent Bonferroni-corrected [21] CIs of 1 − 0.05/14 are reported. If the interval does not include 0, there is evidence that either CheXNeXt or the radiologists are superior in that task.

All statistical analyses were completed in the R environment for statistical computing [22]. The irr package [23] was used to calculate the exact Fleiss’ kappa and Cohen’s kappa. The boot package [24] was used to perform the bootstrap and construct the bootstrap percentile intervals (95% and 99.6%). The ConSpline package [25] was used to estimate the ROC for the radiologists using partial least-squares regression with constrained splines, the pROC package [26] was used to estimate the ROC for the algorithm, and the MESS package [27] was used to calculate the AUC for both the radiologists and CheXNeXt. Figures were created using the ggplot2 [28] and gridExtra [29] packages.

## Results

**Fig 1:**
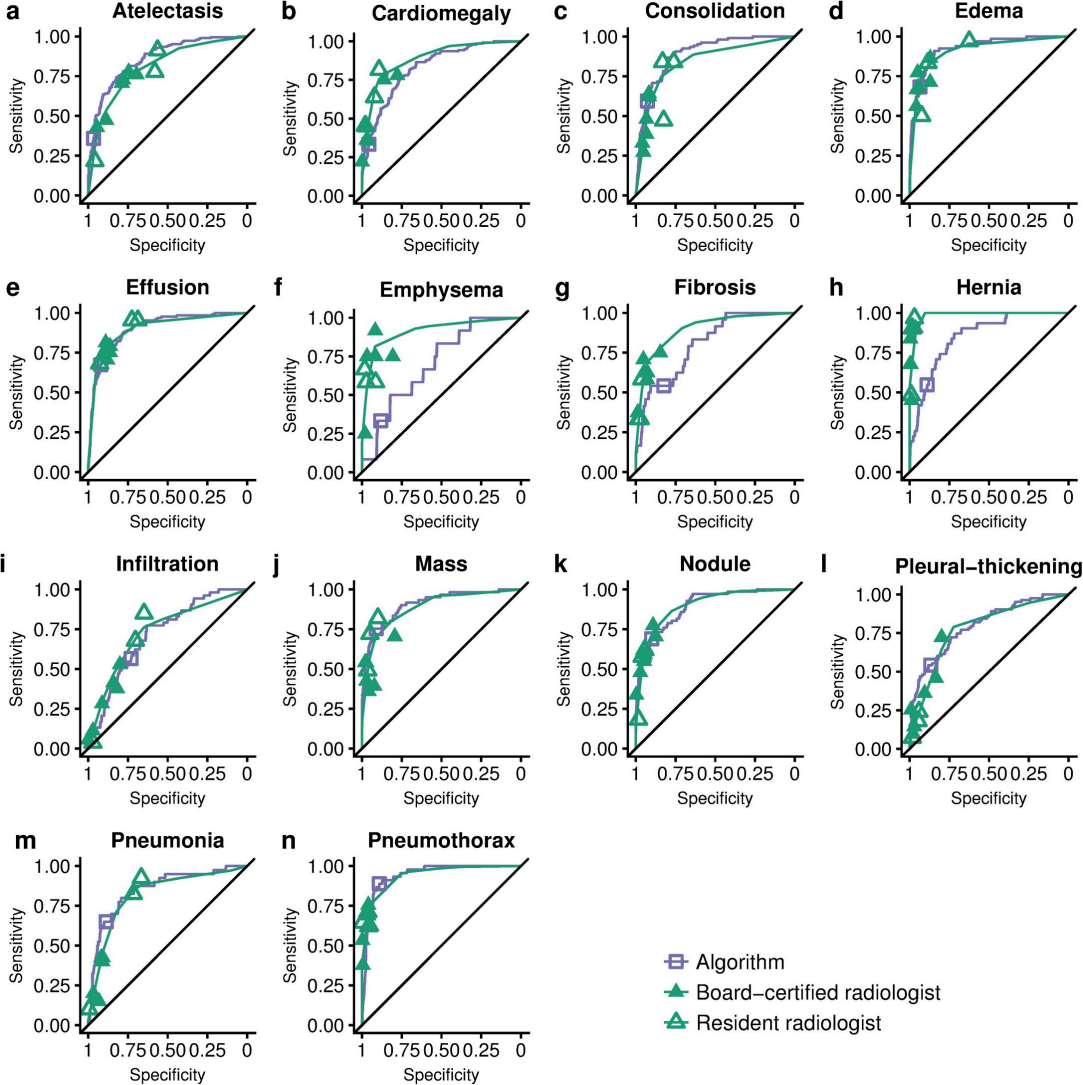
ROC curves of radiologists and algorithm for each pathology on the validation set. Each plot illustrates the ROC curve of the deep learning algorithm (purple) and practicing radiologists (green) on the validation set, on which the majority vote of 3 cardiothoracic subspecialty radiologists served as ground truth. Individual radiologist (specificity, sensitivity) points are also plotted, where the unfilled triangles represent radiology resident performances and the filled triangles represent BC radiologist performances. The ROC curve of the algorithm is generated by varying the discrimination threshold (used to convert the output probabilities to binary predictions). The radiologist ROC curve is estimated by fitting an increasing concave curve to the radiologist operating points (see S1 Appendix). BC, board-certified; ROC, receiver operating characteristic.

The ROC curves for each of the pathologies on the validation set are illustrated in Fig 1, and AUCs with CIs are reported in Table 1; statistically significant differences in AUCs were assessed with the Bonferroni-corrected CI (1 − 0.05/14). The CheXNeXt algorithm performed as well as the radiologists for 10 pathologies and performed better than the radiologists on 1 pathology. It achieved an AUC of 0.862 (95% CI 0.825–0.895) for atelectasis, statistically significantly higher than radiologists' AUC of 0.808 (95% CI 0.777–0.838). The radiologists achieved statistically significantly higher AUC performance on cardiomegaly, emphysema, and hiatal hernia, with AUCs of 0.888 (95% CI 0.863–0.910), 0.911 (95% CI 0.866–0.947), and 0.985 (95% CI 0.974–0.991), respectively, whereas CheXNeXt’s AUCs were 0.831 (95% CI 0.790–0.870), 0.704 (95% CI 0.567–0.833), and 0.851 (95% CI, 0.785–0.909), respectively. There were no statistically significant differences in the AUCs for the other 10 pathologies.

**Table 1:** Radiologists and algorithm AUC with CIs.

Pathology	Radiologists (95% CI)	Algorithm (95% CI)	Algorithm − Radiologists Difference (99.6% CI)^a^	Advantage
Atelectasis	0.808 (0.777 to 0.838)	0.862 (0.825 to 0.895)	0.053 (0.003 to 0.101)	Algorithm
Cardiomegaly	0.888 (0.863 to 0.910)	0.831 (0.790 to 0.870)	−0.057 (−0.113 to −0.007)	Radiologists
Consolidation	0.841 (0.815 to 0.870)	0.893 (0.859 to 0.924)	0.052 (−0.001 to 0.101)	No difference
Edema	0.910 (0.886 to 0.930)	0.924 (0.886 to 0.955)	0.015 (−0.038 to 0.060)	No difference
Effusion	0.900 (0.876 to 0.921)	0.901 (0.868 to 0.930)	0.000 (−0.042 to 0.040)	No difference
Emphysema	0.911 (0.866 to 0.947)	0.704 (0.567 to 0.833)	−0.208 (−0.508 to −0.003)	Radiologists
Fibrosis	0.897 (0.840 to 0.936)	0.806 (0.719 to 0.884)	−0.091 (−0.198 to 0.016)	No difference
Hernia	0.985 (0.974 to 0.991)	0.851 (0.785 to 0.909)	−0.133 (−0.236 to −0.055)	Radiologists
Infiltration	0.734 (0.688 to 0.779)	0.721 (0.651 to 0.786)	−0.013 (−0.107 to 0.067)	No difference
Mass	0.886 (0.856 to 0.913)	0.909 (0.864 to 0.948)	0.024 (−0.041 to 0.080)	No difference
Nodule	0.899 (0.869 to 0.924)	0.894 (0.853 to 0.930)	−0.005 (−0.058 to 0.044)	No difference
Pleural thickening	0.779 (0.740 to 0.809)	0.798 (0.744 to 0.849)	0.019 (−0.056 to 0.094)	No difference
Pneumonia	0.823 (0.779 to 0.856)	0.851 (0.781 to 0.911)	0.028 (−0.087 to 0.125)	No difference
Pneumothorax	0.940 (0.912 to 0.962)	0.944 (0.915 to 0.969)	0.004 (−0.040 to 0.051)	No difference

^a^The AUC difference was calculated as the AUC of the algorithm minus the AUC of the radiologists. To account for multiple hypothesis testing, the Bonferroni-corrected CI (1 − 0.05/14; 99.6%) around the difference was computed.

The nonparametric bootstrap was used to estimate the variability around each of the performance measures; 10,000 bootstrap replicates from the validation set were drawn, and each performance measure was calculated for the algorithm and the radiologists on these same 10,000 bootstrap replicates. This produced a distribution for each estimate, and the 95% bootstrap percentile intervals (2.5th and 97.5th percentiles) are reported.

**Abbreviations:** AUC, area under the receiver operating characteristic curve; CI, confidence interval.

**Fig 2:**
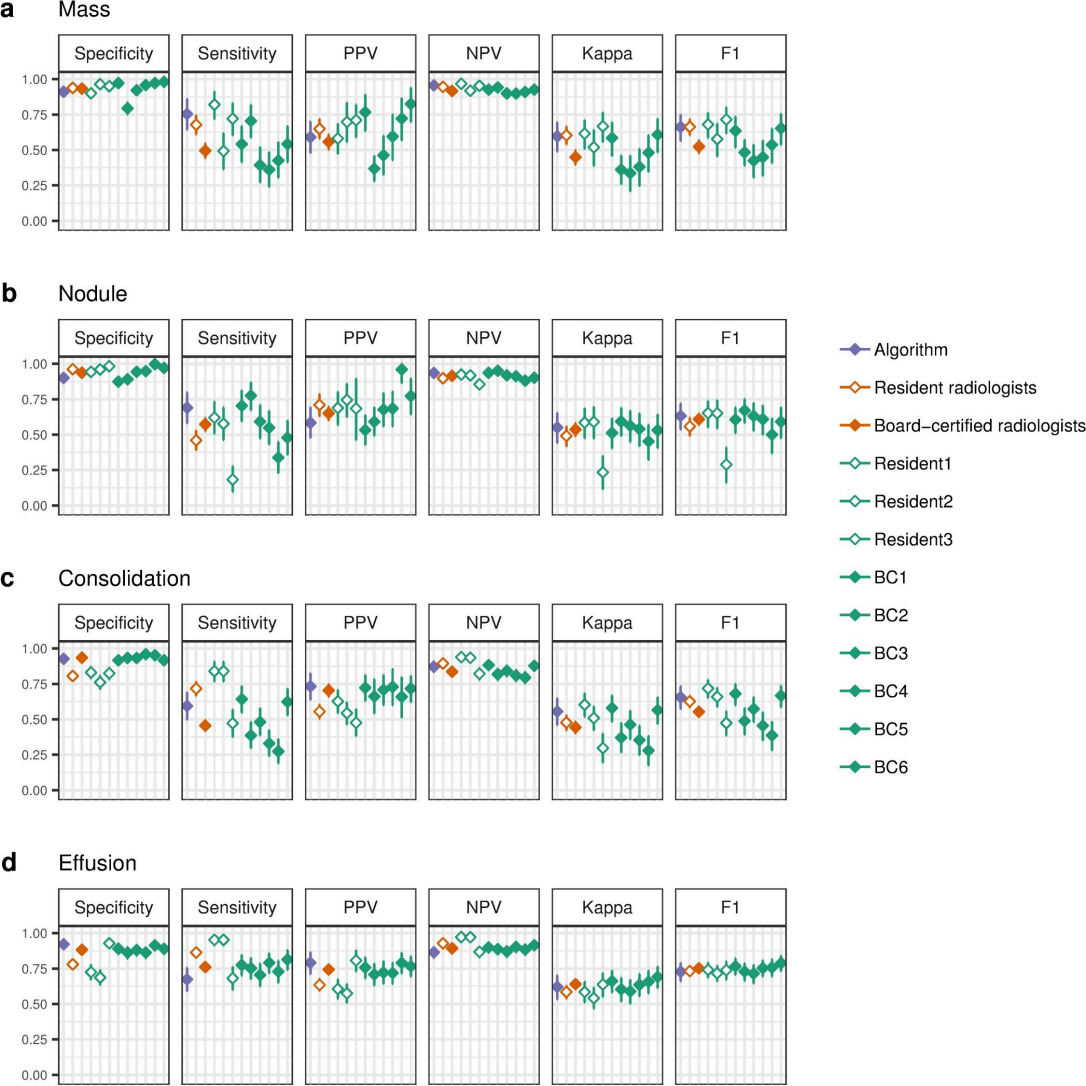
Performance measures of the algorithm and radiologists on the validation set for mass, nodule, consolidation, and effusion. Each plot shows the diagnostic measures of the algorithm (purple diamond), micro-average resident radiologist (unfilled orange diamond), micro-average BC radiologist (filled orange diamond), individual resident radiologists (unfilled green diamond), individual BC radiologists (filled green diamond). Each diamond has a vertical bar denoting the 95% CI of each estimate, computed using 10,000 bootstrap replicates. The ground truth values used to compute each metric were the majority vote of 3 cardiothoracic specialty radiologists on each image in the validation set. Kappa refers to Cohen's Kappa, and F1 denotes the F1 score. BC, board-certified; CI, confidence interval; NPV, negative predictive value; PPV, positive predictive value.

Performance measure results for mass, nodule, consolidation, and effusion are illustrated in Fig 2 (panels a–d), and numerical values for those pathologies are reported in S1 File. The CheXNeXt algorithm detected masses and nodules with sensitivities of 0.754 (95% CI 0.644–0.860) and 0.690 (95% CI 0.581–0.797), respectively, which was higher than the micro-average sensitivities of board-certified radiologists at 0.495 (95% CI 0.443–0.546) and 0.573 (95% CI 0.525–0.619), respectively (Fig 2). CheXNeXt maintained high specificity in both tasks, achieving 0.911 (95% CI 0.880–0.939) in mass detection and 0.900 (95% CI 0.867–0.931) in nodule detection compared with radiologist scores of 0.933 (95% CI 0.922–0.944) and 0.937 (95% CI 0.927–0.947) for mass and nodule, respectively. In identifying consolidation, algorithm specificity was 0.927 (95% CI 0.897–0.954) and sensitivity was 0.594 (95% CI 0.500–0.688), compared with micro-average board-certified radiologist specificity 0.935 (95% CI 0.924–0.946) and sensitivity 0.456 (95% CI 0.418–0.495). The CheXNeXt algorithm detected effusion with a specificity of 0.921 (95% CI 0.889–0.951), higher than micro-average board-certified radiologist specificity of 0.883 (95% CI 0.868–0.898) while achieving a sensitivity of 0.674 (95% CI 0.592–0.754), comparable to micro-average board-certified radiologist sensitivity of 0.761 (95% CI 0.731–0.790). The results for the other 10 pathologies are shown in S1 Fig, and numerical values are provided in S1 File.

The effects of training set prevalence and the relabeling procedure on algorithm performance are illustrated in S2 Table. The algorithm performed significantly worse than radiologists on cardiomegaly, emphysema, and hernia, all of which had low prevalence in the original training set. On pneumonia, fibrosis, and edema, however, the algorithm performed as well as radiologists even though the prevalence of labels in the original training set was low. Our relabeling procedure resulted in an increase in the number of positive labels for every pathology. Using the new labels, the algorithm’s performance improved on 11 pathologies and worsened for 3 pathologies.

The mean proportion correct values with SDs of the algorithm and the radiologists are shown in S3 Table. The algorithm had a mean proportion correct for all pathologies of 0.828 (SD 0.12) compared with 0.675 (SD 0.15) and 0.654 (SD 0.16) for board-certified radiologists and residents, respectively. This indicates that over all 14 pathologies, the algorithm predictions agreed with the cardiothoracic specialist radiologists' findings more often than board-certified general radiologists (on average, 15.3% more often). S4 Table and S5 Table display additional performance and radiologist agreement results.

**Fig 3:**
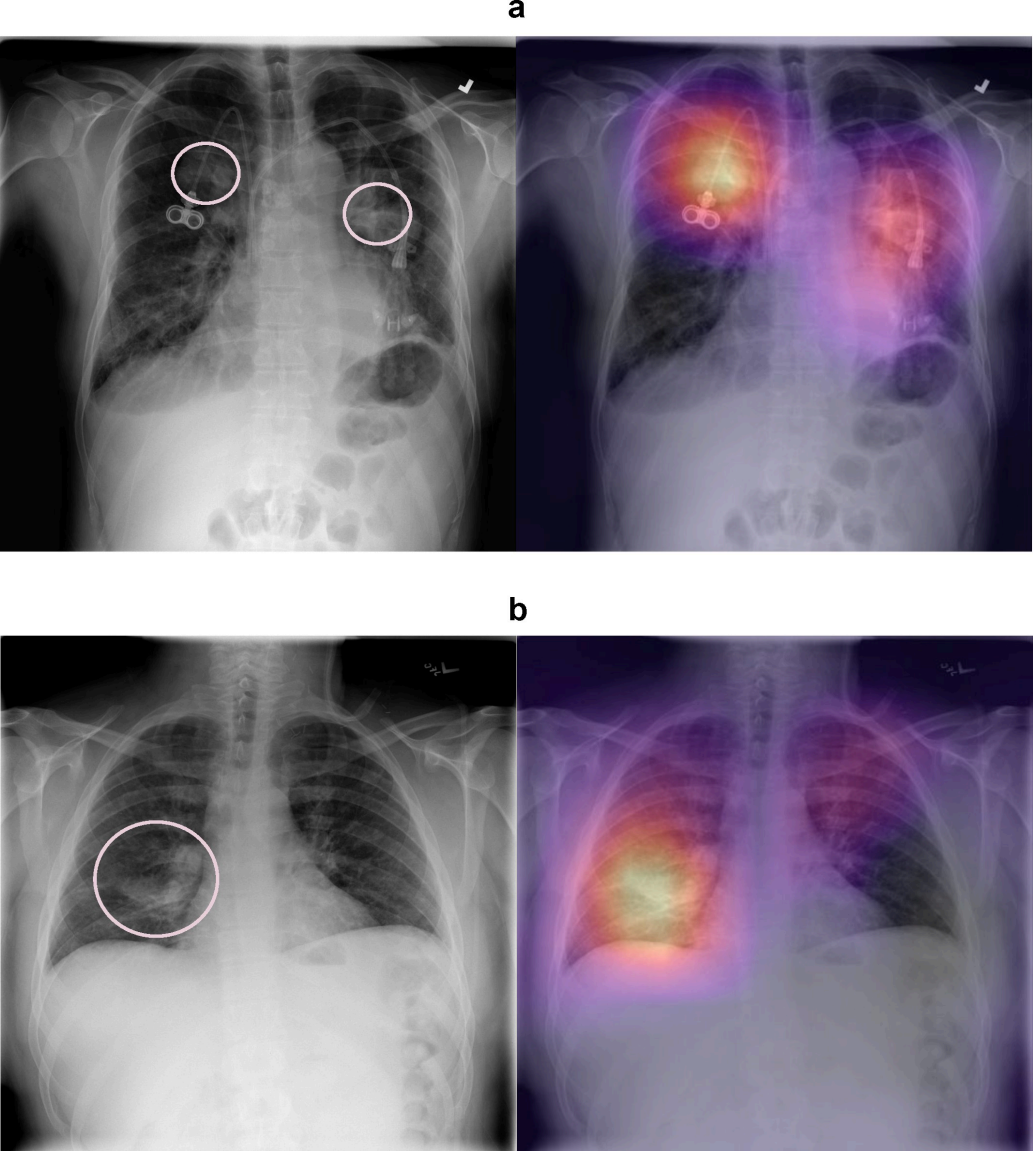
Interpreting network predictions using CAMs. In the normal chest radiograph images (left), the pink arrows and circles highlight the locations of the abnormalities; these indicators were not present when the image was input to the algorithm. (a) Frontal chest radiograph (left) demonstrates 2 upper-lobe pulmonary masses in a patient with both right- and left-sided central venous catheter. The algorithm correctly classified and localized both masses as indicated by the heat maps. (b) Frontal chest radiograph demonstrates airspace opacity in the right lower lobe consistent with pneumonia. The algorithm correctly classified and localized the abnormality. More examples can be found in S2 Fig. CAM, class activation mapping.

The average time for radiologists to complete labeling of 420 chest radiographs was 240 minutes (range 180–300 minutes). The deep learning algorithm labeled the same 420 chest radiographs in 1.5 minutes and produced heat maps highlighting areas of the image that are indicative of a particular pathology in 40 additional seconds. Fig 3 panels a and b show examples of heat maps for different pathologies, and more examples can be found in S2 Fig.

## Discussion

The results presented in this study demonstrate that deep learning can be used to develop algorithms that can automatically detect and localize many pathologies in chest radiographs at a level comparable to practicing radiologists. Clinical integration of this system could allow for a transformation of patient care by decreasing time to diagnosis and increasing access to chest radiograph interpretation.

The potential value of this tool is highlighted by the World Health Organization, which estimates that more than 4 billion people lack access to medical imaging expertise [30]. Even in developed countries with advanced healthcare systems, an automated system to interpret chest radiographs could provide immense utility [31,32]. This algorithm could be used for worklist prioritization, allowing the sickest patients to receive quicker diagnoses and treatment even in hospital settings in which radiologists are not immediately available. Furthermore, experienced radiologists are still subject to human limitations, including fatigue, perceptual biases, and cognitive biases, all of which lead to errors [33–37]. Prior studies suggest that perceptual errors and biases can be reduced by providing feedback on the presence and locations of abnormalities on radiographs to interpreting radiologists [38], a scenario that is well suited for our proposed algorithm.

An additional application for CheXNeXt is screening of tuberculosis and lung cancer, both of which use chest radiography for screening, diagnosis, and management [39–43]. The CheXNeXt algorithm detected both consolidation and pleural effusion, the most common findings for primary tuberculosis, at the level of practicing radiologists. Similarly, CheXNeXt achieved radiologist-level accuracy for both pulmonary nodule and mass detection, a critical task for lung cancer diagnosis, with much higher specificity than previously reported computer-aided detection systems and comparable sensitivity [44–47]. Although chest radiography is not the primary method used to perform lung cancer screening, it is the most common thoracic imaging study in which incidental lung cancers (nodules or masses) are discovered. For example, in a large study of incidentally discovered lung cancers in 593 patients, 71.8% were diagnosed incidentally on chest X-ray and the remaining on computed tomography (CT) scan [48]. This would suggest that, despite the recommendation and widespread use in modernized healthcare environments for the use of screening CT, chest radiographs remain the primary modality by which lung cancer is imaged. Additionally, lung cancers are sometimes diagnosed on chest CT and then identified in retrospect as “missed” on previous chest radiographs. This scenario is not rare and has a considerable medicolegal impact on the field of radiology. Furthermore, the vast majority of the world’s population does not have access to chest CT for lung cancer screening or diagnosis and therefore must rely on the versatile and less resource-intensive chest radiograph for the detection of thoracic pathologies, including lung cancer and tuberculosis. Once clinically validated, an algorithm such as CheXNeXt could have impactful clinical applications in healthcare systems.

While CheXNeXt performed extremely well in comparison to board-certified radiologists on acute diagnoses, it performed poorest in the detection of emphysema and hiatal hernia. The symmetric "global" radiographic appearance in emphysema (symmetric pulmonary overexpansion) may have been more challenging to recognize as opposed to asymmetric "localized" findings such as pulmonary nodule, effusion, or pneumothorax. In addition, hiatal hernia was the least prevalent of all the 14 labels in the training data. These shortcomings could be addressed in the future by obtaining more labeled training data for these pathologies.

Additionally, the sensitivity of board-certified radiologists in the detection of mass was low. To investigate this, we evaluated the sensitivity of the board-certified radiologists and algorithm after grouping the mass and nodule pathology classes as lung lesion (if the label was positive for either nodule or mass, the new label was positive for lung lesion; otherwise, it was negative). Before collapsing these classes, the board-certified radiologists achieved a sensitivity of 0.573 in detecting nodules and 0.495 in detecting masses. After collapsing, the board-certified radiologists achieved a sensitivity of 0.667 in the detection of lung lesions. This indicates that the board-certified radiologists frequently selected the nodule label when the ground truth was mass but did accurately detect a pulmonary lesion. CheXNeXt had higher sensitivities for mass and nodule than board-certified radiologists (0.754 and 0.690, respectively) and maintained a higher sensitivity (0.723) after grouping.

This study has limitations that likely led to a conservative estimate of both radiologist and algorithm performance. First, the radiologists and algorithm only had access to frontal radiographs during reading, and it has been shown that up to 15% of accurate diagnoses require the lateral view [1]. The lack of lateral views in the dataset may limit detection of certain clinical findings such as vertebral body fractures or subtle pleural effusions not detected on frontal views alone; future work may consider utilizing the lateral views when applicable for diagnosis and algorithm development. Second, neither CheXNeXt nor the radiologists were permitted to use patient history or review prior examinations, which has been shown to improve radiologist diagnostic performance in interpreting chest radiographs [49,50]. Third, the images were presented to the radiologists and the CheXNeXt algorithm at a resolution of 1,024 pixels and 512 pixels, respectively, and chest radiographs are usually presented at a resolution of over 2,000 pixels. Fourth, the reference standard was decided by a consensus of cardiothoracic radiologists, and no access to cross-sectional imaging, laboratory, or pathology data was available to determine the reference standard. The comparison to gold standard cases for all pathologies is outside the scope and purpose of this study. Instead, the goal is to evaluate the performance of a deep learning algorithm in diagnostic tasks on radiographs using a retrospective approach based on the interpretations of an expert panel compared with the interpretations of individual nonspecialist radiologists. Finally, consolidation, infiltration, and pneumonia are all manifestations of airspace opacities on chest radiographs yet were provided as distinct labels. While any given radiograph can be marked with one or more of these 3 labels, certain radiographic patterns of airspace opacities are characteristic of pneumonia and, when combined with clinical information, can determine the pneumonia diagnosis specifically. Even in the absence of clinical data, identifying airspace opacity patterns characteristic of pneumonia is useful, particularly in parts of the world where access to expert diagnostics is limited.

This work has additional limitations that should be considered when interpreting the results. This study is limited to evaluation on a dataset from a single institution, so future work will be necessary to address generalizability of these algorithms to datasets from other institutions. Additionally, the experimental design used to assess radiologists in this work does not replicate the clinical environment, so the radiologist performance scores presented in this study may not exactly reflect true performance in a more realistic setting. Specifically, disagreement in chest radiograph interpretation between clinical radiologists has been well described and would not always be interpreted as error in clinical practice, e.g., atelectasis is not always a clinically important observation, particularly if other findings are present. In that way, the labeling task performed by the radiologist readers in this study differs from routine clinical interpretation because in this work, any/all relevant findings in each image were labeled as present no matter the potential clinical significance. Finally, the primary performance metric comparison in this study required estimating the ROC for radiologists. While we assumed symmetry in the specificities and sensitivities, allowing for a better fit, we acknowledge that this is not a perfect comparison, and for this reason, we also provided a comprehensive view of how the algorithm compares to radiologists on 6 other performance metrics (Fig 2 and S1 Fig). All performance metrics and estimates of uncertainty should be taken together to better understand the performance of this algorithm in relation to these practicing radiologists.

## Conclusion

We present CheXNeXt, a deep learning algorithm that performs comparably to practicing board-certified radiologists in the detection of multiple thoracic pathologies in frontal-view chest radiographs. This technology may have the potential to improve healthcare delivery and increase access to chest radiograph expertise for the detection of a variety of acute diseases. Further studies are necessary to determine the feasibility of these outcomes in a prospective clinical setting.

## Supporting information

S1 Fig Performance measures of the algorithm and radiologists on the validation set for all other pathologies. Each plot shows the diagnostic measures of the algorithm (purple diamond), micro-average resident radiologist (unfilled orange diamond), micro-average BC radiologist (filled orange diamond), individual resident radiologists (unfilled green diamond), individual BC radiologists (filled green diamond). Each diamond has a vertical bar denoting the 95% CI of each estimate, computed using 10,000 bootstrap replicates. The ground truth values used to compute each metric were the majority vote of 3 cardiothoracic specialty radiologists on each image in the validation set. Kappa refers to Cohen's Kappa, and F1 denotes the F1 score. BC, board-certified; NPV, negative predictive value; PPV, positive predictive value.
(TIF)

S2 Fig Interpreting network predictions. The left image in each panel is the original radiograph with radiologist annotations (pink ovals) highlighting the abnormality in the radiograph; these indicators were not present when the images were input to the algorithm. The right image in each panel is the localization heatmap output by the algorithm overlaying the original image. (a–b; d–f) The algorithm correctly identified and localized the abnormality as indicated by the heat map. In panel c, the algorithm correctly classified the abnormality, but the heat map indicates that the algorithm incorrectly localized the abnormality and instead focused on the chest tube. (a) Large round mass in the retrocardiac midline containing an air-fluid level consistent with a hiatal hernia. (b) Mass in the right upper lobe. (c) Right-sided pneumothorax and 2 right-sided chest tubes. (d) Right lower lobe airspace opacities consistent with pneumonia. (e) Evidence of edema. (f) Pleural effusion in the right lung base.
(TIF)

S1 Table Summary statistics of training, tuning, and validation datasets. (DOCX)

S2 Table ChestX-ray14 training set label prevalence compared with algorithm performance. (DOCX)

S3 Table Mean proportion correct over all pathologies on the validation set. (DOCX)

S4 Table Inter-rater agreement of the 3 cardiothoracic specialist radiologists on the validation set. (DOCX)

S5 Table ChestX-ray14 label statistics and ChestX-ray14 label agreement with the validation set. (DOCX)

S1 Appendix Supplementary methods. (DOCX)

S1 File Performance measure values of the algorithm and radiologists on the references standard set for all pathologies. The “Resident radiologists” expert refers to the micro-average over the 3 resident radiologists and “BC radiologists” expert refers to the micro-average over the 6 board-certified radiologists. Individual estimates follow.
(XLSX)

## Abbreviations

AUC: area under the receiver operating characteristic curve
CAM: class activation mapping
CI: confidence interval
IRB: International Review Board
NPV: negative predictive value
PPV: positive predictive value
ROC: receiver operating characteristic
