# Supplementary figures and images for: Deep learning for chest radiograph diagnosis: A retrospective comparison of the CheXNeXt algorithm to practicing radiologists
Source: PLoS Med. 2018 Nov 20;15(11):e1002686. doi: 10.1371/journal.pmed.1002686 (PMC6245676; doi:10.1371/journal.pmed.1002686)

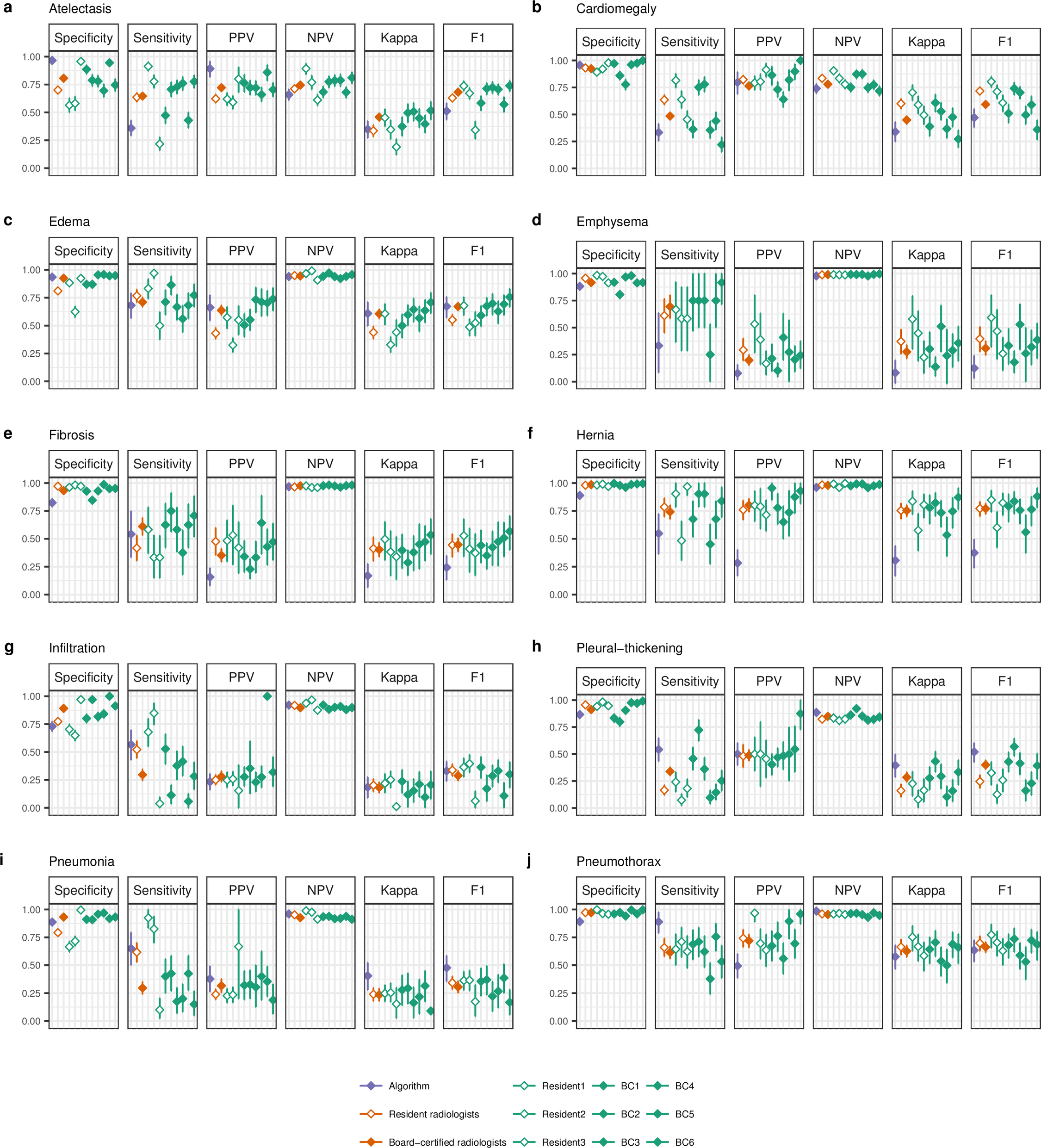

Supplement: S1 Fig — Each plot shows the diagnostic measures of the algorithm (purple diamond), micro-average resident radiologist (unfilled orange diamond), micro-average BC radiologist (filled orange diamond), individual resident radiologists (unfilled green diamond), individual BC radiologists (filled green diamond). Each diamond has a vertical bar denoting the 95% CI of each estimate, computed using 10,000 bootstrap replicates. The ground truth values used to compute each metric were the majority vote of 3 cardiothoracic specialty radiologists on each image in the validation set. Kappa refers to Cohen's Kappa, and F1 denotes the F1 score. BC, board-certified; NPV, negative predictive value; PPV, positive predictive value. (TIF) [file pmed.1002686.s001.tif]

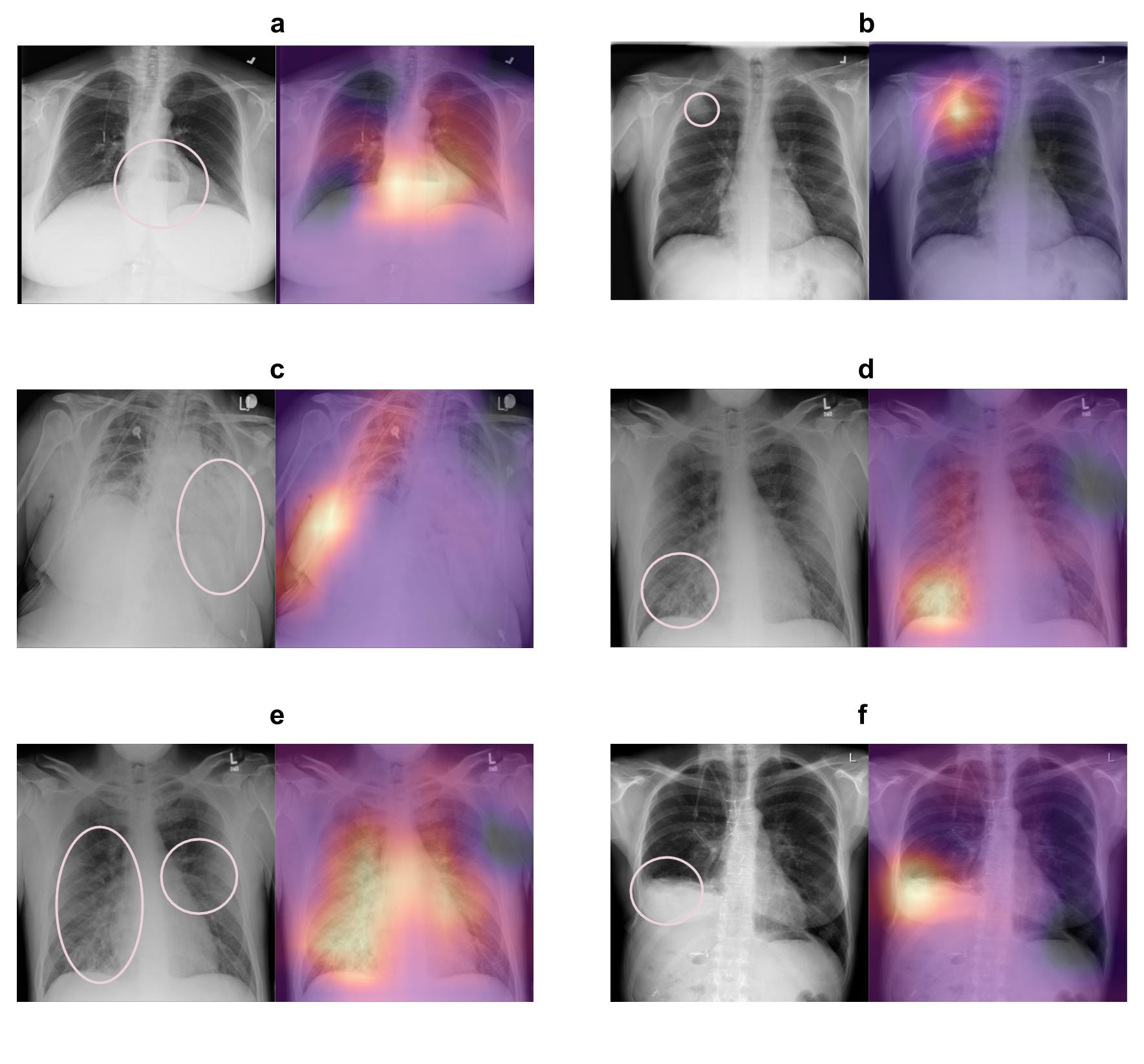

Supplement: S2 Fig — The left image in each panel is the original radiograph with radiologist annotations (pink ovals) highlighting the abnormality in the radiograph; these indicators were not present when the images were input to the algorithm. The right image in each panel is the localization heatmap output by the algorithm overlaying the original image. (a–b; d–f) The algorithm correctly identified and localized the abnormality as indicated by the heat map. In panel c, the algorithm correctly classified the abnormality, but the heat map indicates that the algorithm incorrectly localized the abnormality and instead focused on the chest tube. (a) Large round mass in the retrocardiac midline containing an air-fluid level consistent with a hiatal hernia. (b) Mass in the right upper lobe. (c) Right-sided pneumothorax and 2 right-sided chest tubes. (d) Right lower lobe airspace opacities consistent with pneumonia. (e) Evidence of edema. (f) Pleural effusion in the right lung base. (TIF) [file pmed.1002686.s002.tif]
