# Supplementary material for: Deep learning for chest radiograph diagnosis: A retrospective comparison of the CheXNeXt algorithm to practicing radiologists
Source: PLoS Med. 2018 Nov 20;15(11):e1002686. doi: 10.1371/journal.pmed.1002686 (PMC6245676; doi:10.1371/journal.pmed.1002686)
Supplement: S1 Table — (DOCX) [file pmed.1002686.s003.docx]

**S1 Table.** **Summary Statistics of Training, Tuning, and Validation Datasets**.

| **Pathology** | **Train ChestX-ray14 Labels No. (%)** | **Tuning ChestX-ray14 Labels No. (%)** | **Validation Labels No. (%)** |
| --- | --- | --- | --- |
| Atelectasis | 10053 (10.2) | 671 (10.6) | 184 (43.8) |
| Cardiomegaly | 2293 (2.3) | 110 (1.7) | 141 (33.6) |
| Consolidation | 3869 (3.9) | 262 (4.1) | 106 (25.2) |
| Edema | 1820 (1.8) | 130 (2.0) | 66 (15.7) |
| Effusion | 11326 (11.5) | 752 (11.8) | 129 (30.7) |
| Emphysema | 2011 (2.0) | 135 (2.1) | 12 (2.9) |
| Fibrosis | 1414 (1.4) | 89 (1.4) | 24 (5.7) |
| Hernia | 110 (0.1) | 4 (0.1) | 31 (7.4) |
| Infiltration | 16947 (17.2) | 1186 (18.7) | 53 (12.6) |
| Mass | 4878 (4.9) | 347 (5.5) | 61 (14.5) |
| Nodule | 5437 (5.5) | 374 (5.9) | 71 (16.9) |
| Pleural Thickening | 2802 (2.8) | 175 (2.8) | 83 (19.8) |
| Pneumonia | 1107 (1.1) | 65 (1.0) | 40 (9.5) |
| Pneumothorax | 4360 (4.4) | 237 (3.7) | 45 (10.7) |
| Total No. of Images^a^ | 98637 | 6351 | 420 |
| Total No. of Patients | 28744 | 1672 | 389 |

The training set was used to optimize network parameters, the tuning set was used to compare and choose networks, and the validation set was used to evaluate CheXNeXt and radiologists.

^a^ Numbers do not sum to group totals since each image can have zero or multiple labels. Percentages do not add to 100% for the same reason.
