# Supplementary material for: Deep learning for chest radiograph diagnosis: A retrospective comparison of the CheXNeXt algorithm to practicing radiologists
Source: PLoS Med. 2018 Nov 20;15(11):e1002686. doi: 10.1371/journal.pmed.1002686 (PMC6245676; doi:10.1371/journal.pmed.1002686)
Supplement: S2 Table — (DOCX) [file pmed.1002686.s004.docx]

**S2 Table. ChestX-ray14 Training Set Label Prevalence Compared with Algorithm Performance.**

| **Pathology** | **AUC of algorithm trained on original training set (95% CI)** | **AUC of algorithm trained on relabeled training set (95% CI)** | **Radiologists**  **(95% CI)** | **Original Train ChestX-ray14 Labels No. (%)** | **Relabeled Train ChestX-ray14 Labels No. (%)** |
| --- | --- | --- | --- | --- | --- |
| Atelectasis | 0.843 (0.803,0.878) | 0.862 (0.825,0.895) | 0.808 (0.777,0.838) | 10053 (10.2) | 17487 (17.7) |
| Cardiomegaly | 0.827 (0.784,0.867) | 0.831 (0.79,0.87) | 0.888 (0.863,0.910) | 2293 (2.3) | 7936 (8.0) |
| Consolidation | 0.860 (0.819,0.896) | 0.893 (0.859,0.924) | 0.841 (0.815,0.870) | 3869 (3.9) | 15249 (15.5) |
| Edema | 0.933 (0.901,0.96) | 0.924 (0.886,0.955) | 0.910 (0.886,0.930) | 1820 (1.8) | 11369 (11.5) |
| Effusion | 0.901 (0.869,0.929) | 0.901 (0.868,0.93) | 0.900 (0.876,0.921) | 11326 (11.5) | 19036 (19.3) |
| Emphysema | 0.790 (0.709,0.862) | 0.704 (0.567,0.833) | 0.911 (0.866,0.947) | 2011 (2.0) | 5306 (5.4) |
| Fibrosis | 0.809 (0.705,0.9) | 0.806 (0.719,0.884) | 0.897 (0.840,0.936) | 1414 (1.4) | 10963 (11.1) |
| Hernia | 0.782 (0.704,0.852) | 0.851 (0.785,0.909) | 0.985 (0.974,0.991) | 110 (0.1) | 5051 (5.12) |
| Infiltration | 0.710 (0.636,0.778) | 0.721 (0.651,0.786) | 0.734 (0.688,0.779) | 16947 (17.2) | 22977 (23.3) |
| Mass | 0.880 (0.828,0.926) | 0.909 (0.864,0.948) | 0.886 (0.856,0.913) | 4878 (4.9) | 10410 (10.6) |
| Nodule | 0.882 (0.842,0.92) | 0.894 (0.853,0.93) | 0.899 (0.869,0.924) | 5437 (5.5) | 11627 (11.8) |
| Pleural Thickening | 0.783 (0.731,0.834) | 0.798 (0.744,0.849) | 0.779 (0.740,0.809) | 2802 (2.8) | 11204 (11.4) |
| Pneumonia | 0.774 (0.678,0.858) | 0.851 (0.781,0.911) | 0.823 (0.779,0.856) | 1107 (1.1) | 9838 (10.0) |
| Pneumothorax | 0.917 (0.868,0.956) | 0.944 (0.915,0.969) | 0.940 (0.912,0.962) | 4360 (4.4) | 10417 (10.6) |

The first two columns illustrate the area under the ROC curve (AUC) of the algorithm on the validation set when training on the original training set (first column) and the relabeled training set (second column). The third column shows the estimated AUC of the average radiologist. The fourth column contains the number of cases (and percentage) of each pathology in the training set according to the original ChestX-ray14 labels. The final column contains the number of positive labels from relabeling the training set by computing an “or” (disjunction) between the original labels and ensemble labels. This table contains identical data from S1 Table and Table 1 in the main text.
