# Supplementary material for: Deep learning for chest radiograph diagnosis: A retrospective comparison of the CheXNeXt algorithm to practicing radiologists
Source: PLoS Med. 2018 Nov 20;15(11):e1002686. doi: 10.1371/journal.pmed.1002686 (PMC6245676; doi:10.1371/journal.pmed.1002686)
Supplement: S3 Table — (DOCX) [file pmed.1002686.s005.docx]

**S3 Table. Mean Proportion Correct Over All Pathologies on the Validation Set.**

| Expert | Mean (SD) |
| --- | --- |
| Algorithm | 0.828 (0.12) |
| Resident radiologists | 0.654 (0.16) |
| BC radiologists | 0.675 (0.15) |
| Resident1 | 0.627 (0.18) |
| Resident2 | 0.630 (0.17) |
| Resident3 | 0.706 (0.12) |
| BC1 | 0.677 (0.16) |
| BC2 | 0.634 (0.14) |
| BC3 | 0.658 (0.14) |
| BC4 | 0.688 (0.14) |
| BC5 | 0.701 (0.14) |
| BC6 | 0.690 (0.15) |

To provide an overall estimate of accuracy, the proportion correct was calculated for each image across all 14 pathologies. The mean and standard deviation (SD) of these proportions are reported.
