# Supplementary material for: Deep learning for chest radiograph diagnosis: A retrospective comparison of the CheXNeXt algorithm to practicing radiologists
Source: PLoS Med. 2018 Nov 20;15(11):e1002686. doi: 10.1371/journal.pmed.1002686 (PMC6245676; doi:10.1371/journal.pmed.1002686)
Supplement: S4 Table — (DOCX) [file pmed.1002686.s006.docx]

**S4 Table. Inter-rater Agreement of the 3 Cardiothoracic Specialist Radiologists on the Validation Set.**

| Pathology | Exact Fleiss’ kappa |
| --- | --- |
| Atelectasis | 0.277 |
| Cardiomegaly | 0.399 |
| Consolidation | 0.215 |
| Edema | 0.493 |
| Effusion | 0.481 |
| Emphysema | 0.312 |
| Fibrosis | 0.253 |
| Hernia | 0.614 |
| Infiltration | 0.099 |
| Mass | 0.376 |
| Nodule | 0.489 |
| Pleural Thickening | 0.306 |
| Pneumonia | 0.130 |
| Pneumothorax | 0.474 |

The exact Fleiss’ kappa between the 3 cardiothoracic specialist radiologists on the validation set was computed. This metric assesses the reliability of agreement among a group of raters and accounts for agreement by chance. The maximum kappa score is 1, and a non-positive kappa score is interpreted as no agreement between the raters (except what would be expected by chance).
