# Supplementary material for: Deep learning for chest radiograph diagnosis: A retrospective comparison of the CheXNeXt algorithm to practicing radiologists
Source: PLoS Med. 2018 Nov 20;15(11):e1002686. doi: 10.1371/journal.pmed.1002686 (PMC6245676; doi:10.1371/journal.pmed.1002686)
Supplement: S5 Table — (DOCX) [file pmed.1002686.s007.docx]

**S5 Table. ChestX-ray14 Label Statistics and ChestX-ray14 Label Agreement with the Validation Set.**

| Pathology | No. (%) | Cohen’s kappa |
| --- | --- | --- |
| Atelectasis | 60 (14.3) | 0.237 |
| Cardiomegaly | 50 (11.9) | 0.308 |
| Consolidation | 53 (12.6) | 0.206 |
| Edema | 50 (11.9) | 0.462 |
| Effusion | 53 (12.6) | 0.224 |
| Emphysema | 56 (13.3) | 0.012 |
| Fibrosis | 61 (14.5) | 0.193 |
| Hernia | 50 (11.9) | 0.715 |
| Infiltration | 59 (14.0) | 0.094 |
| Mass | 60 (14.3) | 0.488 |
| Nodule | 54 (12.9) | 0.279 |
| Pleural Thickening | 58 (13.8) | 0.263 |
| Pneumonia | 50 (11.9) | 0.180 |
| Pneumothorax | 55 (13.1) | 0.569 |

The number (No.) column denotes the number of cases for each pathology according to the ChestX-ray14 labels (as well as percentage of the entire validation set). To estimate the quality of the ChestX-ray14 labels, we computed the Cohen’s kappa between the validation set labels (the majority vote of the cardiothoracic specialist radiologists) and the ChestX-ray14 labels. The Cohen’s kappa coefficient measures the inter-rater agreement among two raters and accounts for agreement by chance. The metric attains its maximum value at 1 (complete agreement), and minimum value at 0 (no agreement except what would be expected by chance).
