# Supplementary material for: Deep learning for chest radiograph diagnosis: A retrospective comparison of the CheXNeXt algorithm to practicing radiologists
Source: PLoS Med. 2018 Nov 20;15(11):e1002686. doi: 10.1371/journal.pmed.1002686 (PMC6245676; doi:10.1371/journal.pmed.1002686)
Supplement: S1 Appendix — (DOCX) [file pmed.1002686.s008.docx]

**Supplementary Methods**

**Architecture and Hyperparameter Selection**

All hyperparameter choices were made by examining performance on the tuning set. We first found that increasing the input scale from 224x224 to 512x512 improved the performance of our models on the tuning set. The batch size of 8 was chosen as it was the maximum size which could fit in memory on a 6GB GPU. We found that augmenting the data during training using random horizontal improved performance, but random rotations did not. We selected the optimal starting learning rate from the range of values 1e-2, 1e-3, 1e-4, 1e-5 with the adaptive annealing scheme described in the text. We also found that using weighted loss led to better overall performance than unweighted loss. We did not experiment with any form of regularization besides early stopping and data augmentation, which would be worth investigating in future work.

**Selecting Thresholds for Inference**

In order to convert the network probabilities to binary predictions at inference time, we chose a threshold for every pathology such that the network outputs positive for that pathology if the probability output by the network is greater than that threshold, and negative otherwise. For each pathology, we selected the threshold by maximizing the F1 score, the harmonic mean between precision and recall, on the tuning set. The exact thresholds determined were:

Atelectasis: 0.69288576

Cardiomegaly: 0.75185317

Consolidation: 0.74413335

Edema: 0.86919022

Effusion: 0.7312175

Emphysema: 0.86045086

Fibrosis: 0.78941596

Hernia: 0.83123577

Infiltration: 0.63273901

Mass: 0.75189936

Nodule: 0.71835887

Pleural Thickening: 0.72703892

Pneumonia: 0.83110636

Pneumothorax: 0.75975639

**ROC Fitting Example**

Load the ConSpline and MESS libraries

library(ConSpline)

library(MESS)

## Loading required package: coneproj

Create a data frame with x = 1 - specificity and y = sensitivity.

Here is an example.

spec <- c(0.8700565,0.8700565,0.9548023,0.9576271,0.9463277,0.9491525,0.8841808,0.6242938,0.9237288)
sens <- c(0.7121212,0.8636364,0.6666667,0.5606061,0.6818182,0.7727273,0.8333333,0.9696970,0.5000000)
df <- data.frame(x = 1-spec,y = sens)
plot(df, xlim=c(0,1), ylim=c(0,1))


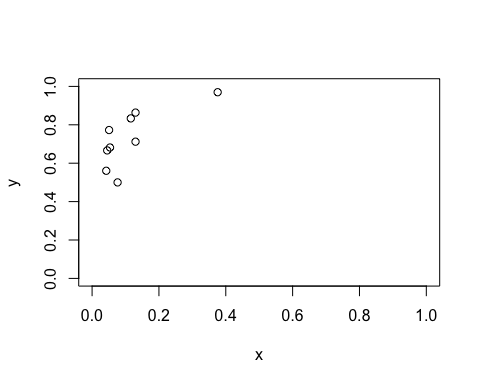


Assume symmetry to get a closer fit and place anchors in the corners at (0,0) and (1,1).

rx <- rbind(df,data.frame(x = 1 - df$y, y = 1-df$x))
 rx <- rbind(c(0,0),rx,c(1,1))
plot(rx, xlim=c(0,1), ylim=c(0,1))


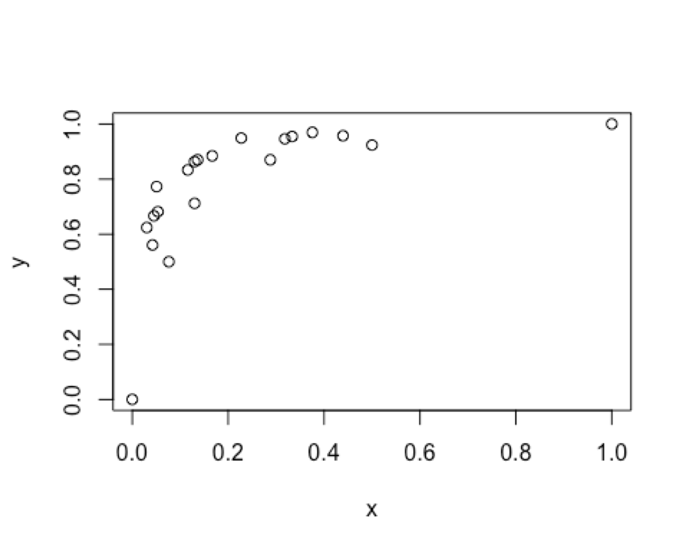


Fit the ROC model specifying a concave increasing curve (type=7).

knots <- seq(0,1,0.05)
mod <- conspline(rx$y, rx$x, type=7, knots = knots)
rxx <- rx
rxx$y <- mod$muhat
# sort by x
rxx <- rxx[order(rxx[,1]),]
plot(rxx, col="red", xlab="1-specificity", ylab="sensitivity")
lines(rxx, col="red")
points(1-spec,sens, pch=24)
legend(x="bottomright",
 legend=c("Experts","Estimated ROC"),
 col=c("black","red"),
 pch=c(24,1),
 lty=c(0,1))


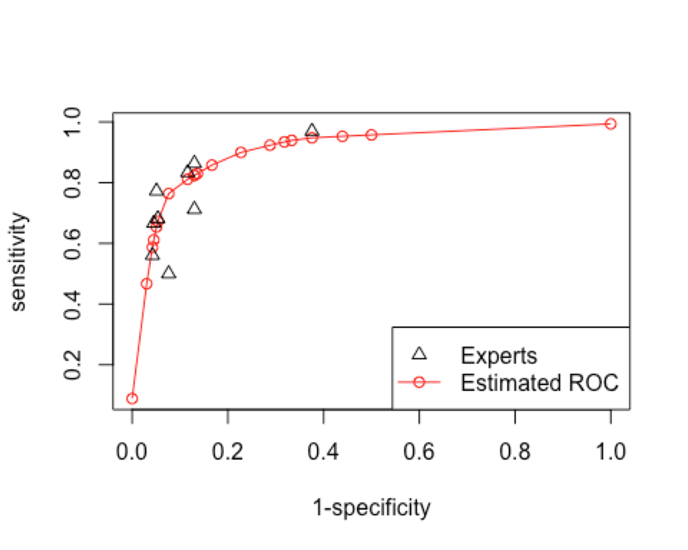


Calculate the AUC

MESS::auc(rxx$x,rxx$y)

## [1] 0.9087489
